# Effect and cerebral mechanism of acupuncture treatment for functional constipation: study protocol for a randomized controlled clinical trial

**DOI:** 10.1186/s13063-019-3410-8

**Published:** 2019-05-24

**Authors:** Tao Yin, Zhaoxuan He, Peihong Ma, Likai Hou, Li Chen, Kunnan Xie, Zilei Tian, Fumin Wang, Jing Xiong, Yi Yang, Ruirui Sun, Fang Zeng

**Affiliations:** 1 Acupuncture and Tuina School/The 3rd Teaching Hospital, Chengdu University of Traditional Chinese Medicine, 37# Shierqiao Road, Chengdu, 610075 Sichuan China; 2 Acupuncture-Brain Research Center, Chengdu University of Traditional Chinese Medicine, Chengdu, Sichuan China; 3 School of Administration, Chengdu University of Traditional Chinese Medicine, Chengdu, Sichuan China

**Keywords:** Acupuncture, Functional constipation, Central mechanism, Functional magnetic resonance imaging, Positron emission tomography-computed tomography, Multimodal neuroimaging, Clinical trial, Protocol

## Abstract

**Background:**

Acupuncture is effective in functional constipation (FC) treatment, but the central mechanism has not been well investigated. This trial will combine functional magnetic resonance imaging (fMRI) and positron emission tomography-computed tomography (PET-CT) to investigate the potential central mechanism of acupuncture treatment for FC.

**Methods:**

This is a multimodal neuroimaging randomized controlled trial. In total, 140 FC patients will be randomly allocated into four groups: the verum acupuncture group; the sham acupuncture group; the PEG 4000 group; and the waiting-list group. This trial will include a two-week baseline period and a two-week treatment period. Patients will receive 10 sessions of acupuncture, sham acupuncture, PEG 4000, or no intervention during the treatment period. The stool diary, Cleveland Constipation Score (CCS), Patient Assessment of Constipation Symptom (PAC-SYM), and Patient Assessment of Constipation Quality of Life Questionnaire (PAC-QoL) will be used to assess the clinical efficacy of different interventions. The MRI and PET-CT scans will be performed to detect cerebral functional changes in 15 patients in each group at baseline and at the end of treatment/waiting. Multimodal imaging data will be associated with clinical data to investigate possible correlation between brain activity changes elicited by different interventions and symptoms improvement.

**Discussion:**

We hypothesize that acupuncture can treat FC through normalizing the pathological alteration of the cerebral activity. The results of this trial will allow us to re-testify the therapeutic effects of acupuncture treating for FC and to investigate the potential central mechanism of acupuncture treatment for FC from direct (cerebral glucose metabolism) and indirect (contrast of oxyhemoglobin and deoxyhemoglobin) approaches.

**Trial registration:**

Chinese Clinical Trial Registry, ChiCTR1800016658. Registered on 14 June 2018.

**Electronic supplementary material:**

The online version of this article (10.1186/s13063-019-3410-8) contains supplementary material, which is available to authorized users.

## Background

Functional constipation (FC) is a common functional gastrointestinal disease (FGID) which is characterized by persistently difficult and low frequent defecation, often accompanied with straining and incomplete sensation but without organic abnormality of the lower abdomen [1, 2]. As a worldwide public health issue, the prevalence of FC is in the range of 12–19% in North America [3] and is about 14% in Asia [4], 19.2% in Europe, and 19.7% in Oceania [5]. Although FC is not life-threatening, it seriously influences FC patients’ quality of life (QoL) and has high medical costs [6–8]. The general measures to manage FC contain lifestyle modification and medication. Lifestyle modification, such as dietary fiber intake and physical activity, are recommended to improve bowel movement, whereas the efficacy still remains uncertain and the strength of recommendation is weak [9, 10]. Medicines, which are conventionally prescribed to FC patients, such as osmotic laxatives, stimulant laxatives, gastrointestinal prokinetic agents, and spasmolytic, are reported with definite efficacy, but the side effects of the majority of medicines, such as electrolyte disturbances, dehydration, bowel cramps, and esophageal obstruction, cannot be neglected [10]. As a result, seeking an effective complementary and alternative therapy with few side effects for FC has drawn the attention of both physicians and patients.

Acupuncture, as a widely used complementary and alternative therapy originating from ancient China, has been used to manage constipation for thousands of years. In recent years, many high-quality randomized controlled trials (RCTs) have also demonstrated the efficacy of acupuncture treating for FC [11–15]. For example, Liu et al. found that electro-acupuncture could significantly increase the frequency of spontaneous defecation with rare adverse events in chronic severe constipation [12]. Our recent study also identified that manual acupuncture at Tianshu (ST25) and Shangjuxu (ST37) acupoints were as effective as Mosapride intake in improving spontaneous bowel movements (SBMs) in chronic FC [16]. With the clinical effects being successfully acknowledged, the mechanism of acupuncture treating for FC has also become a focus in acupuncture research [17, 18]. Several studies have found that acupuncture may treat FC by promoting the gastrointestinal motility [19], modulating peripheral gastrointestinal hormones [20], and keeping the balance of excitatory and inhibitory neurons in the enteric nervous system (ENS) [21]. However, the central integration [22, 23], an essential node of acupuncture treatment for FC, has rarely been explored. In previous studies, we found that the therapeutic effects of acupuncture for functional dyspepsia (FD) [24] and functional diarrhea [25] were closely related to regulating the cerebral activity of disease-related regions. Whether the effects of acupuncture on FC are correlated with modulating cerebral activity remains uncertain and is worthy of further investigation.

Therefore, we designed this multimodal neuroimaging trial, aiming to: (1) re-testify the therapeutic effects of acupuncture treating for FC by comparing verum acupuncture with sham acupuncture and Polyethylene glycol (PEG) 4000; (2) explore the potential central mechanism of acupuncture treatment for FC by combining functional magnetic resonance imaging (fMRI) and positron emission tomography-computed tomography (PET-CT); (3) and investigate the possible correlation of brain activity changes elicited by different interventions with symptom improvements.

## Method and Design

### Study design

**Fig. 1:**
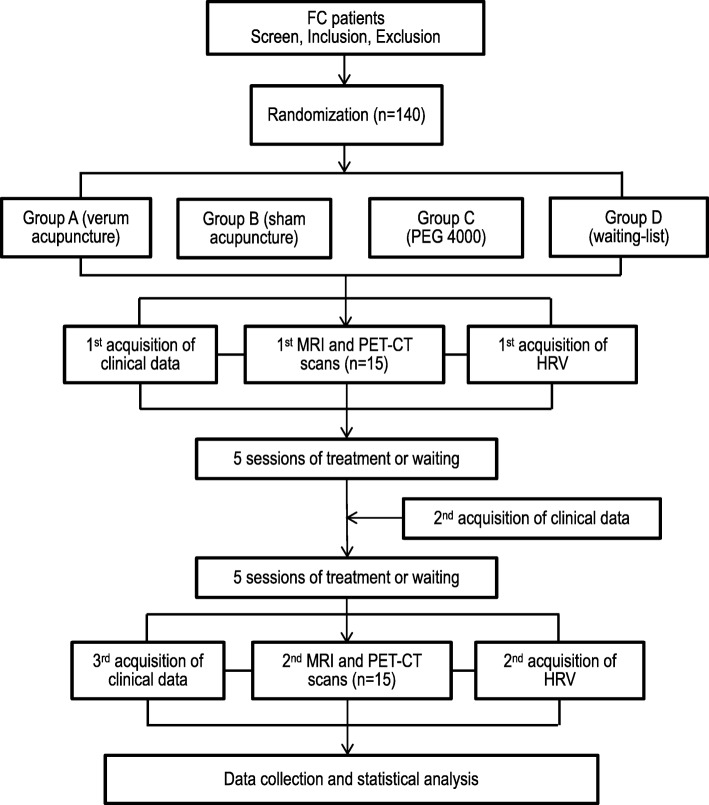
Study schedule: 140 eligible patients will be randomly allocated into four groups with a 1:1:1:1 ratio. Fifteen patients in each group will be randomly selected to undergo MRI and PET-CT scans. Imaging data and HRV will be collected at baseline and at the end of the two-week treatment/waiting . Clinical data will be acquired at three timepoints: baseline; mid-point; and the end of treatment/waiting. FC functional constipation, PEG polyethylene glycol, fMRI functional magnetic resonance imaging, PET-CT Positron emission tomography-computed tomography, HRV heart rate variability

This is a multimodal neuroimaging RCT. A total of 140 eligible FC patients diagnosed with the Rome IV diagnostic criteria [26] will be recruited and randomized to four groups with a 1:1:1:1 ratio. Fifteen participants in each group will be randomly selected to undergo both MRI and PET-CT scans. Outcome assessments will be performed at three timepoints: baseline; mid-point; and the end of treatment/waiting (see Fig. 1 for a detailed study schedule).

**Fig. 2:**
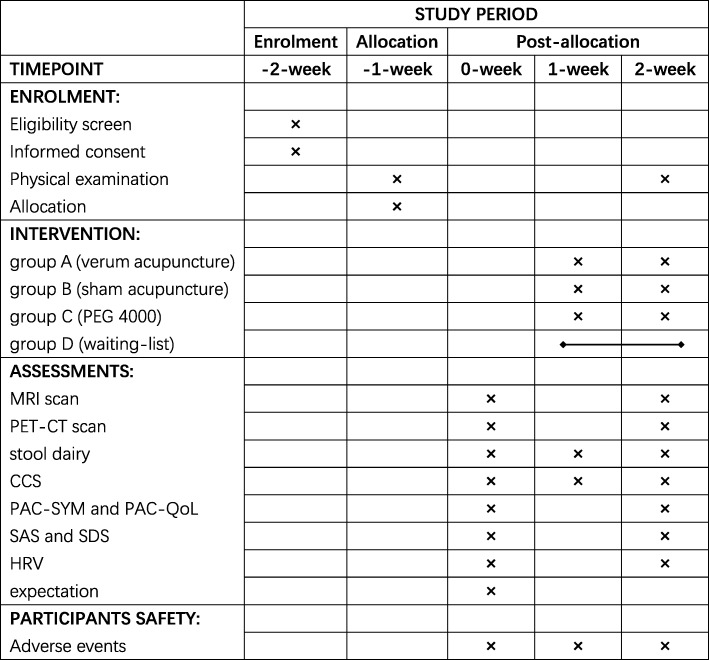
Standard Protocol Items: Recommendations for Interventional Trials (SPIRIT) schedule of the trial. This is a multimodal neuroimaging randomized controlled trial which includes a two-week baseline period and a two-week treatment period. In the baseline period, recruited patients will be screened according to the inclusion criteria and exclusion criteria; then, eligible FC patients will give informed consent and receive a physical examination. After allocation, the patients will receive 10 sessions of acupuncture, sham acupuncture, PEG 4000, or no intervention during the treatment period. The outcome assessments, MRI, and PET-CT scans are performed at baseline and at the end of treatment/waiting. In addition, the stool diary and CCS will also be evaluated at the mid-point (at the end of the first week of treatment/waiting). Physical examination (blood routine test and blood biochemical test) will be performed at the end of treatment to evaluate risks correlated with acupuncture and PEG4000. Adverse events will be recorded in the CRFs at any time during treatment. PEG polyethylene glycol, fMRI functional magnetic resonance imaging, PET-CT positron emission tomography-computed tomography, CCS Cleveland Constipation Score, PAC-SYM Patient Assessment of Constipation Symptom, PAC-QoL Patient Assessment of Constipation Quality of Life Questionnaire, SAS Self-rating Anxiety Scale, SDS Self-rating Depression Scale, HRV heart rate variability

This trial is reported in accordance with the Standard Protocol Items: Recommendations for Intervention Trials (SPIRIT) guidelines [27] (Fig. 2, Additional file 1) and follows the principles of the CONSORT and STRICTA [28].

### Participants

In the present trial, all potential FC patients matching the diagnostic criteria will undergo a physical examination including transabdominal ultrasound, dynamic electrocardiogram, routine blood test, routine urine test, routine stool test, and blood biochemical test (ALT, AST, BUN, Scr). Digestive physicians from the First Teaching Hospital of Chengdu University of Traditional Chinese Medicine (CDUTCM) will make the final diagnosis to include patients.

### Recruitment strategy

Participants will be recruited from the outpatient clinic in the digestive department of the First Teaching Hospital of CDUTCM and campus of CDUTCM from September 2018 to June 2020. The recruitment strategies include delivering leaflets at the outpatient clinic and campus, posting advertisements in billboards, and distributing news at the website of Acupuncture-Brain Research Center, CDUTCM (http://cdutcm.edu.cn/), and our WeChat public account.

### Diagnostic criteria

Rome IV Diagnostic Criteria for FC [26] which promulgated in 2016.

### Inclusion criteria

Patients fulfilling the following six items will be included: (1) matching the diagnostic criteria of FC according to Rome IV; (2) aged 20–40 years; (3) right-handed; (4) not having taken a gastrointestinal prokinetic agent or laxative at least 15 days before enrollment; (5) not participating in any other clinical trials in the past three months; and (6) having signed informed consent.

### Exclusion criteria

Patients matching any of the following five items will be excluded: (1) having any organic or metabolic diseases of the digestive, hematopoietic, endocrine, or immune systems or having any other severe primary diseases; (2) experiencing severe depressive or anxiety disorder; (3) having serious headache, migraine, or having a history of head trauma or gastrointestinal surgery; (4) women having severe dysmenorrhea, being in pregnancy or lactation, or intending to pregnant in the following six months; or (5) having contraindications of MRI or PET-CT scans such as claustrophobia, tattoo, or implanted ferromagnetic metal.

### Sample size

According to a previous systematic review [29] that compared the therapeutic effects of acupuncture, lactulose, and sham acupuncture for FC, the mean weekly improvement in SBMs of 2.46, 2.17, and 1.76 were found in the verum acupuncture, lactulose, and sham acupuncture group, respectively. We anticipate a mean weekly SBM improvement of 2.5 in the verum acupuncture group, 2.2 in the PEG 4000 group, 1.8 in the sham acupuncture group, and 0.9 in the waiting-list control group. Considering α = 0.05, 1-β = 0.8, and a standard deviation of 2.0, a sample size of 128 is needed, with a drop-out rate of 10%; a total of 140 FC patients will be finally recruited.

Based on previous neuroimaging studies [30, 31], 12–15 individuals in each group is the reasonable sample size for stable cerebral responses. Therefore, in this trial, 15 participants randomly selected from each group will be scanned by MRI and PET-CT.

### Randomization

Randomization will be carried out in two steps. First, 140 patients will be allocated into four group equally with simple concealed randomization; second, 15 patients will be randomly selected from each group to undergo MRI and PET-CT scans. To avoid bias caused by researchers’ subjective factors, randomization will be implemented by a third-party professional statistician using computer-generated randomization digital table which is created by SAS 9.2 (SAS Institute Inc., Cary, NC, USA). When participant-recruiting staff decides to recruit an eligible FC patient, they will send the patient’s name, gender, age, and telephone numbers to this statistician via a short message. The randomized result will then be put into an opaque envelope and delivered to the acupuncturists.

### Blinding

It is difficult to blind acupuncturists and patients in this trial for different types of interventions. However, it is feasible to confuse outcome assessors and statistical analysts. Patients are told that they will receive one of three effective interventions randomized after enrolment; during the acupuncture treatment, patients in groups A and group B will be separated into cubicles to refrain from communication. Outcome assessors and statistical analysts will be blind to the procedure and results of randomization, group allocation, and intervention.

### Control

We choose conventional medicine (PEG 4000) as a positive control to evaluate the therapeutic effects of acupuncture and select the sham acupuncture as a control to re-identify the specific effects of acupoints. To preclude the interference of self-healing tendency of FC, a waiting-list group is also designed.

### Intervention

#### Group A

**Fig. 3:**
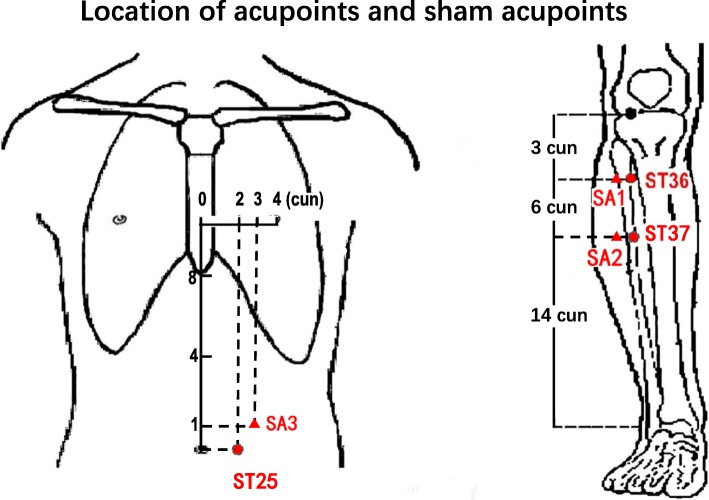
Locations of acupoints and sham acupoints (SA). ST36 (Zusanli), on the anterior lateral side of the shank, 3 *cun* below ST35 (Dubi), one horizontally placed finger distance lateral to the anterior border of the tibia. ST37 (Shangjuxu), on the anterior lateral side of the shank, 6 *cun* below ST35 (Dubi), one horizontally placed finger distance lateral to the anterior border of the tibia. ST25 (Tianshu), on the middle portion of the abdomen, 2 *cun* lateral to the center of the navel. SA1, 1 *cun* outside of Zusanli (ST36) (between the Stomach Meridian and the Gallbladder Meridian), SA2, 1 *cun* outside of Shangjuxu (ST37) (between the Stomach Meridian and the Gallbladder Meridian), and SA3, 1 *cun* outside and above of Tianshu (ST25) (between Stomach Meridian and Spleen Meridian)

Patients in this group will receive manual acupuncture at six verum acupoints (VA) (bilateral Zusanli [ST36], Tianshu [ST25], and Shangjuxu [ST37] (Fig. 3)) with disposable sterile filiform needles (0.25 × 40 mm, Huatuo Medical Instrument Co., Ltd., China). These acupoints have been proven effective and crucial in managing gastrointestinal disorders [16, 32]. Needles will be perpendicularly inserted into acupoints at a depth of 20–30 mm after skin disinfection using alcohol; acupuncturists will then bi-directionally twist needles by 90°–180°, lifting and thrusting needles with the amplitude of 3–5 mm for 1–1.5 Hz to induce *deqi* sensation (including soreness, numbness, distention, and heaviness). After the *deqi* sensation is attained, needles will be retained at the acupoints for 30 min. During the 30 min, the above procedures will be manipulated for 10–15 s every 10 min to maintain the *deqi* sensation. Patients will receive a total of 10 sessions of acupuncture in two weeks with five sessions per week (once a day for five days, followed by a two-day interval).

#### Group B

Patients in group B will receive 10 sessions of manual acupuncture at six sham acupoints (SA) (Fig. 3). Patients in this group will undergo an acupuncture procedure similar to the patients in group A, but no needle manipulation is performed after needle insertion and *deqi* sensation is not required.

All the acupuncture manipulation will be performed by two licensed acupuncturists with at least three years of clinical experience.

#### Group C

Patients assigned to this group will receive conventional medication, the PEG, which has been recommended in some guidelines and has been widely used in the digestive department for its definite efficacy and relatively few side effects [9, 33–35]. Patients will take 10 mg PEG 4000 powder (forlax©) (Ipsen Pharma, France) dissolved with 200–250 mL water once a day before bedtime for 10 days over a period of two weeks. In order to be consistent with the process of acupuncture treatment, oral medicine will also be taken over five consecutive days followed by a two-day interval. After treatment, patients will be required to return any untaken drugs to monitor the adherence.

#### Group D

During the treatment period, no intervention will be performed on this group; however, it has been promised that these participants will receive 10 sessions of acupuncture therapy at the end of the trial, similar to the participants in group A.

Participants with fainting, infection, severe diarrhea, dehydration, or other severe adverse events should be discontinued from treatment and processed immediately. During the treatment period, participants are usually not allowed to use concomitant care and interventions. However, if required, participants will be permitted to use extra osmotic laxatives; the type and dosage of medication used should be recorded in the case report form (CRF). If patients take concomitant care and interventions more than three times in the treatment period, they will be defined as non-adherent and will not be enrolled into the statistical analysis.

### Multimodal data acquisition

#### MRI data acquisition

The morning after an overnight fast, participants will undergo a MRI scan with a 3.0-T MR scanner (Siemens AG, Germany) at Huaxi Magnetic Resonance Research Center, West China Hospital of Sichuan University, Chengdu, China. The scanning procedure contains a localizer, a high-resolution three-dimensional T1-weighted imaging (3D-T1WI), a blood oxygenation level-dependent fMRI (BOLD-fMRI) and a diffusion tensor imaging (DTI) sequence. The 3D-T1WI scanning parameters will be as follows: repetition time (TR)/echo time (TE) = 1900/2.26 ms; slice thickness = 1 mm; slice number = 30; matrix size = 128 × 128; and field of view (FOV) = 256 × 256 mm. The BOLD-fMRI scanning parameters will be as follows: TR/TE = 2000/30 ms; flip angle = 90°; slice number = 30; matrix size = 128 × 128; FOV = 240 × 240 mm; slice thickness = 5 mm; and total volume = 240. The DTI data will be acquired with the following parameters: FOV = 240 × 240 mm; TR/TE = 6800/93 ms; matrix size = 128 × 128; and slice thickness = 3 mm with no gap. Two diffusion-weighted sequences were acquired using gradient values b = 1000 s/mm^2^ and b = 0 with the diffusion-sensitizing gradients applied in 64 non-collinear directions.

#### PET-CT data acquisition

The PET-CT scan will be performed after the MRI scan at the PET-CT center of Sichuan Provincial People’s Hospital, Chengdu, China. The scan will be executed with the Biograph Duo BGO scanner (Siemens AG, Germany) and fluorine-18-fluorodeoxyglucose (^18^F-FDG, radiochemical purity > 95%, 0.11 mCi/kg) will be selected as the tracer. In the scanning period, CT images will be acquired firstly with the parameters as follows: voltage = 120 KV; current = 120 mAs; matrix size = 256 × 256; magnification = 2×; and slice thickness = 3 mm. Then, PET performed with the parameters as follows: bed = 1; model = 3D; slice thickness = 3 mm; slice interval = 2.5 mm; matrix size = 256 × 256; scanning time = 8 min; and total counts = 3 × 10^9^. See Additional file 2 for the detailed MRI and PET-CT scanning process.

### Outcomes

#### Stool diary

The stool diary contains three domains: weekly SBMs; stool feature; and straining sensation. The stool feature is measured by the Bristol Stool Form Scale (BSFS) [36], which classifies the form of human feces into seven types from separate hard lumps (type 1) to entirely liquid (type 7). Straining sensation measured in the range of 0–3 represents the intensity of difficulty during defecation, “0” means no difficulty, “1” means slight difficulty, “2” means medium difficulty, and “3” means much difficulty and assisted measures are needed for defecation.

#### Cleveland Constipation Score (CCS)

The CCS [37] is a designated scale to evaluate patients’ gastrointestinal or anorectal symptoms. The CCS is composed of eight items: defecation frequency; straining feeling; incomplete feeling; abdominal pain; defecating time; assisted measures to defecation; unsuccessful defecation frequency in 24 h; and course of constipation. Each item is scored in the range of 0–4 or 0–2; the summation is in the range of 0–30. A higher score indicates more severe symptoms of constipation.

#### Patient Assessment of Constipation Symptom (PAC-SYM) and Patient Assessment of Constipation Quality of Life Questionnaire (PAC-QoL)

The PAC-SYM [38] is a self-rating scale to assess patients’ constipation symptoms in the last two weeks. It involves three aspects of symptoms (stool symptoms, rectal symptoms, and abdomen symptoms) and each symptom is scored in the range of 0–4 according to severity (“0” means not at all severe, “4” means very severe).

The PAC-QoL [39] is intended to reflect the QoL of FC patients in the last two weeks. It embodies seven dimensions with a total of 28 questions involving physical discomfort, social discomfort, psychological discomfort, anxiety and concern, and satisfaction in daily life. There are five options for each question in the range of 1–5 (“1” indicates no discomfort or feeling very satisfied, “5” indicates extreme severity and always appears or feeling very dissatisfied). A higher score of the PAC-SYM and PAC-QoL suggests more severe symptoms, more discomfort, less satisfaction, and poorer QoL.

#### Self-rating Anxiety Scale (SAS) and Self-rating Depression Scale (SDS)

Anxiety and depression are emotional disorders commonly accompanied by FGIDs [40]. Hence, the SAS [41] and SDS [42] are used to assess the emotional health of FC patients.

In addition, the heart rate variability (HRV) will be measured by dynamic electrocardiogram to evaluate the activity of the autonomic nervous system of FC patients [14].

The above outcomes will be assessed at baseline and at the end of treatment/waiting. In addition, the stool diary and CCS will also be evaluated at mid-point. The expectation for efficacy in each patient will be evaluated at baseline.

All the outcome assessments will be performed by independent outcome assessors. These assessors are trained before participating in this trial and blinded to the randomization.

### Data management

Clinical data will be managed with printed and electronic CRFs. Only outcome assessors have access to CRFs and will perform double-data entry. The Evidence-based Medicine Center of the CDUTCM will be responsible for monitoring the study and data every three months and will make the final decision to terminate the trial.

### Data analysis

#### Clinical data analysis

The clinical data will be analyzed using SPSS 20.0 statistics software (IBM Co., Armonk, NY, USA) based on the per-protocol (PP) principle. Continuous variables on normal distribution will be described with means and standard deviation (SD). Continuous variables conforming to a skewed distribution will be described with median and inter-quartile range. Categorical variables will be described with percentages (%).

Clinical data from the four groups will be compared using one-way ANOVA; statistical analysis between two groups (group A and B, group A and C, group A and D) will be performed using Dunnett’s test. The comparison between baseline and end of treatment in each group will be carried out with a paired samples *t*-test. The clinical outcome stool diary and CCS scores will be compared at three timepoints using the repeated measures ANOVA. Clinical data on skewed distribution will be compared using a non-parametric test. Categorical variables will be compared using the *χ*^2^ test or Fisher’s exact test. The statistical significance threshold is set at 0.05 with the two-sided test.

#### BOLD-fMRI data analysis

The BOLD-fMRI data will be processed and analyzed using SPM12 software (http://www.fil.ion.ucl.ac.uk/spm/) working on MATLAB 2013b (Mathworks, Inc., USA). The main analytical methods for cerebral responses of different interventions contain regional homogeneity (ReHo), amplitude of low frequency fluctuation (ALFF), and seed-based functional connectivity based on the results of ReHo and ALFF.

#### PET-CT data analysis

The SPM12 software will be applied to analyze the PET-CT data based on MATLAB 2013b. We will measure the whole brain radioactivity to present cerebral glucose metabolism and reflect the cerebral activity directly.

Furthermore, the results of BOLD-fMRI and PET-CT will be mutually verified and the imaging data will be associated with clinical data to investigate the possible correlation of brain activity changes elicited by different interventions with symptom improvements.

### Patient safety

In order to evaluate the risks caused by acupuncture and PEG4000, the blood routine test and blood biochemical test will be fulfilled again at the end of treatment. Adverse events such as bleeding, fainting, pain, infection, diarrhea, or other severe events will be recorded in the CRFs in detail and monitored by the Ethics Committees.

## Discussion

### The possibility of acupuncture modulating the abnormal cerebral activity to treat FC

The harmonious bidirectional interaction between the brain and the gut is a necessary prerequisite for retaining normal physiological function and homeostatic state of the gastrointestinal tract [43, 44]. Disharmony of the brain–gut axis might be a critical pathogenesis of FGIDs [26, 45]. Previous neuroimaging studies have found that the functional and/or structural abnormalities of the brain might be the highlighted pathological characteristics of FGIDs [46, 47]. For example, a systematic review of neuroimaging studies on FD found that abnormality of the pain and salience network was a key feature of FD [48]. Another review of irritable bowel syndrome (IBS) also reported that the changes occurring in the descending pain modulation system and the autonomic nervous system were relevant for IBS’s pathophysiology [49]. Furthermore, a resting-state fMRI study [50] performed on FC patients also found that brain regions implicated in emotional, somatic, and sensory processing, and motor control (such as supplementary motor area, dorsal anterior cingulate cortex) might contribute to the progression of FC.

Moreover, studies performed on individuals with FD [24], IBS [51], functional diarrhea [25], Crohn’s disease [52], and healthy controls [53] have found that having influence on the brain activity might be an important reason for modulating effects of acupuncture on gastrointestinal function. For example, our previous study [54] demonstrated that puncturing at ST36 could evoke homeostatic afferent processing network of FD patients, which was confirmed as a pivotal network of acupuncture treating for FD in another study [24]. Therefore, we hypothesize that acupuncture could treat FC through normalizing the pathological alteration of the brain activity.

#### Multimodal neuroimaging technology is an appropriate approach to explore the central mechanism of acupuncture

Multimodal neuroimaging technology refers to combining two or more datasets acquired with different imaging instruments. It has the merits of high spatiotemporal resolution and comprehensive physical and physiological sensitivities [55]. BOLD-fMRI and PET-CT are two of the most widely used neuroimaging technologies in acupuncture-neuroimaging studies [56]. ^18^F-FDG is the most commonly used radiotracer in cognitive and neuroscience research [57–59]. The collaborative application of BOLD-fMRI and ^18^F-FDG PET-CT can prove the structural and functional images with high spatiotemporal and functional resolution [60], which could deliver more details of the brain and explain the central mechanism of acupuncture effects from both direct (cerebral glucose metabolism) and indirect (contrast of oxyhemoglobin and deoxyhemoglobin) approaches.

Meanwhile, brain activity changes caused by acupuncture are not isolated. The application of multimodal neuroimaging technologies and multi-dimensional analysis strategies will bring an appropriate approach to expound the central integration of acupuncture, not only from fractional brain regions but also from functional networks. Last, but not least, cross-validation of the BOLD-fMRI data and ^18^F-FDG PET-CT data will effectively improve the reliability and repeatability of the results. Therefore, in the present trial, we combine these two neuroimaging techniques for the first time to explore the central effects of acupuncture for FC.

### Quality control is the guarantee of the reliability and repeatability of study results

To improve the reliability and repeatability of study results, strict quality control must be enforced. In this trial, quality control will be strengthened not only from participants’ enrolment and acupuncture manipulation [61, 62] but also from multimodal imaging data acquisition and fusion.

Considering the dynamic variability of brain function, imaging data of different modalities should be acquired with a short time interval in a study [55]. Thus, in this trial, MRI and PET-CT data will be acquired on the morning of the same day with an overnight fast. To avoid the interference of unstable physiological and psychological factors, participants will be asked to maintain their regular lifestyle and avoid staying up late and ingesting alcohol and caffeine during the 24 h before the scans. Moreover, the mental state of each participant will be evaluated using SAS and SDS on the morning before scanning. Furthermore, the scanning process will be taken by the same technician with the same scanner; standardized guidance will be given to participants. As for the analysis of multimodal imaging data, BOLD-MRI and PET-CT data will be separately collected and analyzed but interpreted together in the same template space to cross-verify the results.

In conclusion, acupuncture is an effective intervention to manage FC, but the central mechanism is still unclear. This trial is the first multimodal neuroimaging study which combines MRI and PET-CT to explore the potential underlying mechanism of acupuncture treatment for FC with high-quality control.

## Trial status

This trial was registered on 14 June 2018 (registration number ChiCTR1800016658, protocol version number F2.0). The trial is currently in the stage of recruiting patients. The first patient was included on 9 October 2018. To date, 26 patients have been included.

## Additional files

Additional file 1: SPIRIT 2013 Checklist: Recommended items to address in a clinical trial protocol and related documents*. (PDF 121 kb)

Additional file 2: The detailed procedure of PET-CT and MRI scans. (PDF 72 kb)

## Abbreviations

^18^F-FDG: Fluorine-18 fluorodeoxyglucose
3D-T1WI: Three-dimensional T1-weighted imaging
ALFF: Amplitude of low frequency fluctuation
BOLD-fMRI: Blood oxygenation level-dependent fMRI
BSFS: Bristol Stool Form Scale
CCS: Cleveland Constipation Score
CDUTCM: Chengdu University of Traditional Chinese Medicine
CRF: Case report forms
DTI: Diffusion tensor imaging
ENS: enteric nervous system
FC: Functional constipation
FD: Functional dyspepsia
FGID: Functional gastrointestinal disease
fMRI: Functional magnetic resonance imaging
HRV: Heart rate variability
IBS: Irritable bowel syndrome
PAC-QoL: Patient Assessment of Constipation Quality of Life Questionnaire
PAC-SYM: Patient Assessment of Constipation Symptom
PEG: Polyethylene glycol
PET-CT: Positron emission tomography-computed tomography
PP: Per protocol
QoL: Quality of life
RCTs: Randomized controlled trials
ReHo: Regional homogeneity
SA: Sham acupoints
SAS: Self-rating Anxiety Scale
SBMs: Spontaneous bowel movements
SD: Standard deviation
SDS: Self-rating Depression Scale
SPIRIT: Standard Protocol Items Recommendations for Intervention Trials
VA: Verum acupoints
