# Supplementary material for: Effect and cerebral mechanism of acupuncture treatment for functional constipation: study protocol for a randomized controlled clinical trial
Source: Trials. 2019 May 24;20:283. doi: 10.1186/s13063-019-3410-8 (PMC6534837; doi:10.1186/s13063-019-3410-8)
Supplement: Supplementary file 2 — The detailed procedure of PET-CT and MRI scans. (PDF 72 kb) [file 13063_2019_3410_MOESM2_ESM.pdf]

Additional file 2. The detailed procedure of PET-CT and MRI scans

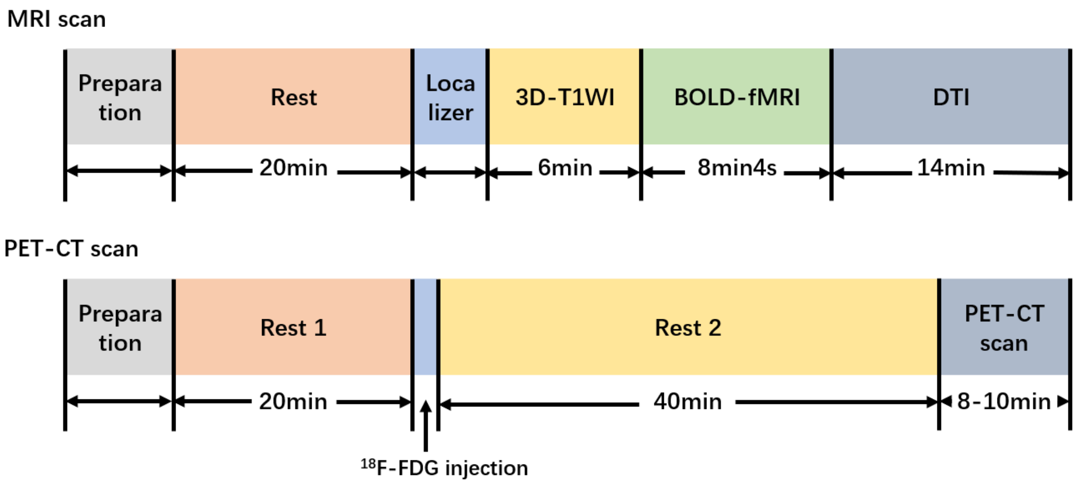

MRI and PET-CT scans will be performed in the morning of the same day with an overnight fast. MRI scan contains 3 steps: (1) preparation (remove metals on the body, put on comfortable clothes and learn scanning precautions carefully); (2) laying supine on the scanning bed with eyes blindfolded and ears plugged for a 20 min rest; (3) undergoing MRI scan with 3 sequences: 3-dimensional T1-weighted imaging(3D-T1WI), blood oxygenation level-dependent fMRI (BOLD-fMRI), and Diffusion Tensor Imaging (DTI). The PET-CT scan will be implemented after MRI scan. the detailed procedure of PET-CT scan embodies 5 steps: (1) preparation (examination of weight, height, and blood sugar, learn scanning precautions carefully); (2) having a rest in a quiet and dark room for 20 min; (3)injection with ^{18}F-FDG (0.11 mCi/kg) via the back of right hand; (4) having a 40min rest; (5) undergoing PET-CT scan for 8-10 min with eyes blindfolded and ears plugged.

a. 3D-T1WI: 3-dimensional T1-weighted imaging; BOLD-fMRI: blood oxygenation level-dependent fMRI; DTI: Diffusion Tensor Imaging; 18F-FDG: Fluorine-18 Fluorodeoxyglucose.
